# A Woman with Vaginal Bleeding and an Intrauterine Device

**DOI:** 10.5811/westjem.2016.5.30482

**Published:** 2016-06-22

**Authors:** Zachary D.W. Dezman, Sarah Sommerkamp

**Affiliations:** University of Maryland, Baltimore, Department of Emergency Medicine, Baltimore, Maryland

## CASE

**Figure 1:**
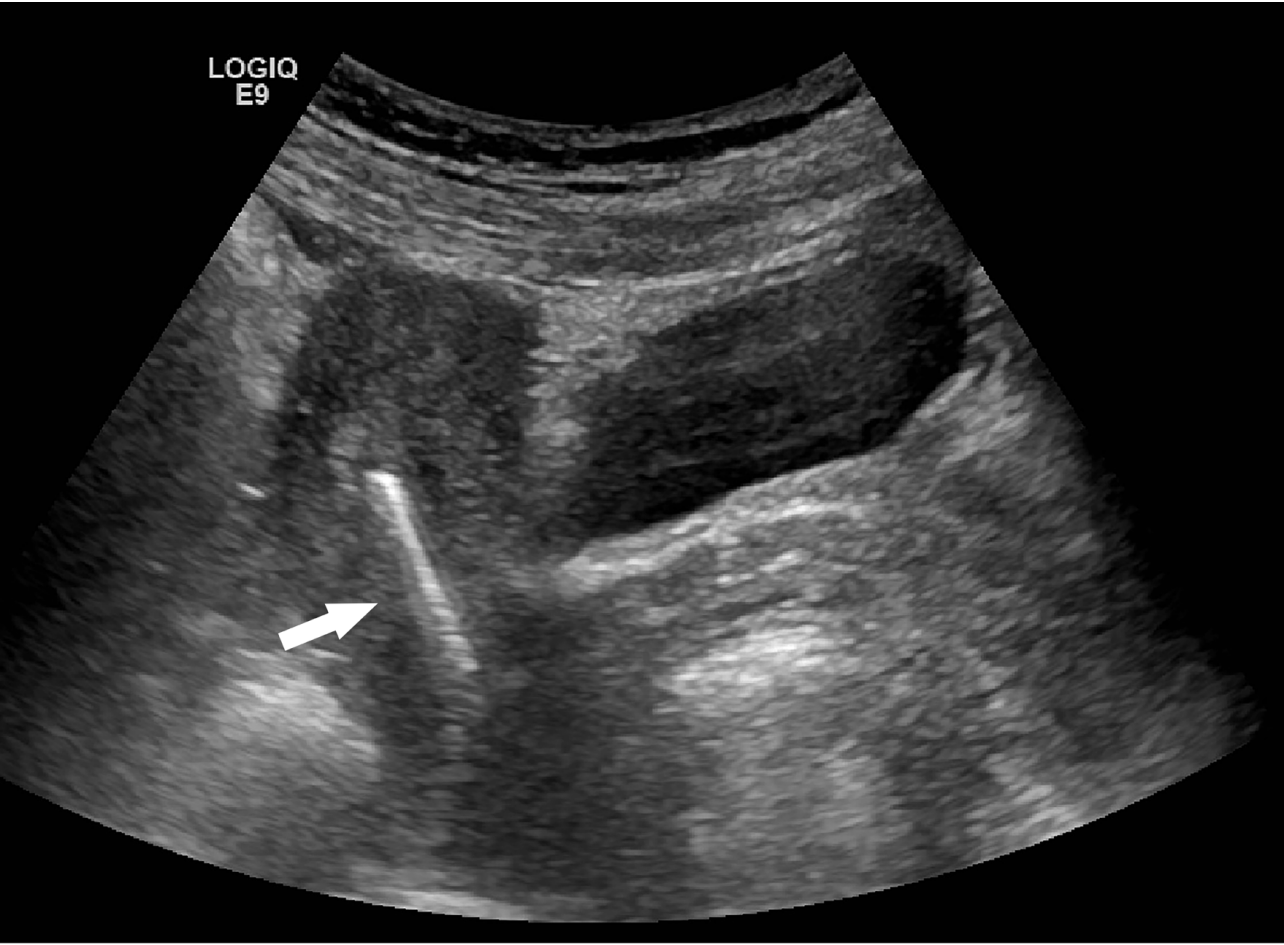
Transvaginal, sagittal sonogram demonstrating an intrauterine device in the left horn (bright white bar, see arrow).

**Figure 2:**
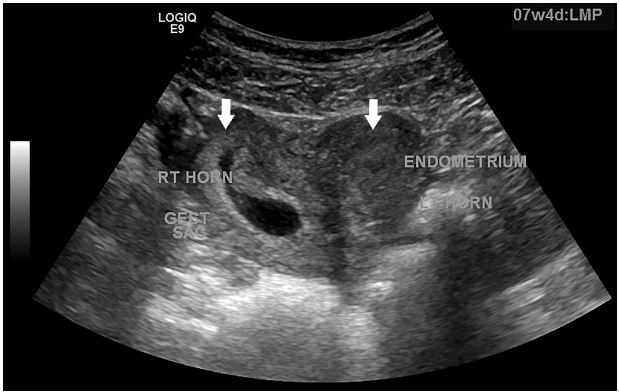
Transvaginal, coronal sonogram showing the classic heart-shape with an intrauterine pregnancy with gestational sac in the right horn (see arrow).

A sexually active 35-year old woman presented to the emergency department with intermittent vaginal spotting and pelvic cramping over the preceding four weeks. She had an intrauterine device (IUD) placed three months prior and has never been pregnant. The threads of the IUD and a small amount of blood coming from the cervix were seen on pelvic exam. Laboratory testing revealed a β-human chorionic gonadotropin level of 70,000 mIU/mL. Pelvic ultrasound imaging showed the IUD (Figure 1) and a viable intrauterine pregnancy (IUP, Figure 2).

## DIAGNOSIS

### Failure of an IUD in a bicornuate uterus

IUDs are generally a reliable method of contraception, with a pregnancy prevention rate of approximately 99.8%.^1^ However, IUD failure can be seen with uterine malformations: uterine septum, didelphys, and bicornuate uterus, all of which arise from a failure of the Mullerian ducts to fuse in-utero. The incidence of these is approximately 0.4%, and they are frequently found incidentally during pregnancy or delivery.^2^ Case studies have reported the successful placement and prevention of pregnancy using IUDs in a bicornuate uterus, though it is recommended that an IUD be placed in each uterine horn. These malformations often present with symptoms consistent with an ectopic pregnancy, an important differential diagnosis. These malformations decrease the effective volume of the uterus, increasing the risk of recurrent fetal loss, fetal malformations, and uterine rupture (as early as 10 weeks gestation).^3^ A pregnancy in the presence of an IUD should alert the physician to further evaluate the patient for a uterine malformation.
